# PARP targeting counteracts gliomagenesis through induction of mitotic catastrophe and aggravation of deficiency in homologous recombination in PTEN-mutant glioma

**DOI:** 10.18632/oncotarget.2993

**Published:** 2014-12-11

**Authors:** Jara Majuelos-Melguizo, María Isabel Rodríguez, Laura López-Jiménez, Jose M. Rodríguez-Vargas, Juan M. Martí Martín-Consuegra, Santiago Serrano-Sáenz, Julie Gavard, J Mariano Ruiz de Almodóvar, F Javier Oliver

**Affiliations:** ^1^ Instituto de Parasitología y Biomedicina López Neyra, CSIC, Granada, Spain; ^2^ IBIMER, Centro de Investigación Biomédica, Universidad de Granada, Spain; ^3^ CNRS, UMR8104, Paris, France

**Keywords:** BUBR1, Mitotic catastrophe, Glioblastoma, Homologous recombination, PARP1, PTEN, genomic instability, EGFR

## Abstract

Glioblastoma multiforme (GBM) is the most common primary brain tumour in adults and one of the most aggressive cancers. PARP-1 is a nuclear protein involved in multiple facets of DNA repair and transcriptional regulation. In this study we dissected the action of PARP inhibition in different GBM cell lines with either functional or mutated PTEN that confers resistance to diverse therapies. In PTEN mutant cells, PARP inhibition induced a severe genomic instability, exacerbated homologous recombination repair (HR) deficiency and down-regulated the Spindle Assembly Checkpoint (SAC) factor BUBR1, leading to mitotic catastrophe (MC). EGFR gene amplification also represents a signature of genetic abnormality in GBM. To more effectively target GBM cells, co-treatment with a PARP inhibitor and an EGFR blocker, erlotinib, resulted in a strong suppression of ERK1/2 activation and *in vivo* the combined effect elicited a robust reduction in tumour development. In conclusion, PARP inhibition targets PTEN-deficient GBM cells through accentuation of SAC repression and aggravation of HR deficiency, leading to the induction of genomic instability and eventually deriving to mitotic catastrophe (MC); the inhibition of PARP and co-treatment with an inhibitor of pro-survival pathways strongly retarded *in vivo* gliomagenesis.

## INTRODUCTION

Glioblastoma multiforme (GBM) are lethal brain tumours, highly resistant to therapy. An important improvement in therapeutic response came from the use of the alkylating agent temozolomide (TMZ) in combination with ionizing radiation (IR). Amongst the huge number of genetic alterations that populate the GBM genomic landscape, five genetic changes dominate: loss of tumour suppressor and aging biomarker (Ink4a), acute renal failure (Arf), cellular tumour antigen p53, or phosphatase and tensin homolog on chromosome 10 (PTEN); and amplification of Epidermal Growth Factor Receptor (EGFR).

Loss of PTEN is a very prominent event during gliomagenesis, occurring in about 36% of GBMs [1-3]. PTEN is a lipid phosphatase with a canonical role in turning-off the phosphatidylinositol 3-kinase (PI3K)-Akt-1 signalling pathway; hence, loss of PTEN has oncogenic consequences during gliomagenesis [4]. In addition, it is becoming increasingly clear that PTEN has novel nuclear functions, including transcriptional regulation of the RAD51 gene, whose product is essential for homologous recombination (HR) repair of DNA breaks [5].

The nuclear protein PARP-1, known to function as a DNA damage sensor and to play a role in various DNA repair pathways, has recently been implicated in a broad variety of cellular functions, including transcriptional regulation [6, 7]. PARP inhibitors exhibit antitumour activity in part due to their ability to induce synthetic lethality in cells deficient for homologous recombination repair [8-12] and also in triple negative breast cancer cells [13, 14]. Up to date, the studies on GBM and PARP inhibitors have focused on the use of these small molecules as radio or chemo-potentiators [15-17]. In this study, we report that primary glioma stem-like cells are targeted in their ability to form neurospheres by PARP inhibitors; moreover, mutant PTEN GBM cells are also sensitive to PARP inhibitors by increasing genomic instability leading to impaired G2/M arrest and MC. Additionally, PARP inhibition strongly intensified HR deficiency in PTEN mutant cells, that had already compromised HR [18-20] and repressed the Spindle Assembly Checkpoint protein BUBR1. Moreover, the combination of PARP inhibition with an upstream blocker of pro-survival signalling pathways arising from the EGFR, the EGFR inhibitor erlotinib, induced a dramatic reduction in tumour growth in an orthotopic mouse model. Thus, taking advantage of PARP inhibitor-induced cell death in PTEN-mutant glioma cells prone to genomic instability, and disabling survival pathways through EGFR and PARP inhibition, could be therapeutically exploited in the treatment of this malignant tumour.

## RESULTS

### PJ34 and olaparib interfere with neurospheres formation in primary glioma cells and impacts differently on cell viability in PTEN wild type and PTEN-mutant glioma cells

**Figure 1:**
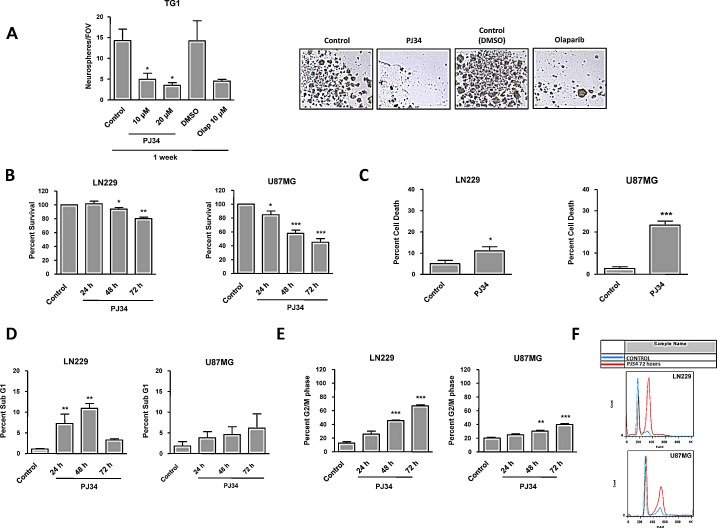
Cell viability in PTEN wt LN229 and PTEN mut U87MG glioblastoma cell lines after treatment with PARP inhibitor (PJ34 20 μM if not detailed) A. Neurosphere Formation Assay (NFA) of GSCs TG1 treated with PJ34 or olaparib. The drug was refreshed the three first days of the experiment. After one week, neurosphere counting (10 fields of view per condition) was performed. *p < 0.05 *versus* control group by t-test. B. Viability analysis by MTT assay of glioblastoma cells treated with PJ34 for 24, 48 and 72 hours. Data were normalized and expressed as a percentage of the control. *p < 0.05, **p < 0.01, ***p < 0.001 *versus* control group by t-test. C. Trypan blue intake counting was in order to check cell death. D. Apoptosis activation was determined 24, 48 and 72 hours after the treatment. SubG1 fraction was analysed by flow cytometry following staining with PI. **p < 0.01 *versus* control group by t-test. E. Cell cycle arrest was determined 24, 48 and 72 hours after the treatment. G2/M fraction was analysed by flow cytometry following staining with PI. **p < 0.01, ***p < 0.001 *versus* control group by t-test. F. Cell cycle profiles referring to control and PJ34 72 hours in both cell lines are represented. Data are represented as mean ± SEM of 3 independent experiments.

As a first approach to analyze the potential of PARP inhibition (PARPi) as monotherapy against GBM we evaluated self-renewal capability, which is a marker of stemness in GSCs, using neurospheres formation assay in primary patient-derived PTEN-proficient GSCs TG1 [21] with two different PARP inhibitors: PJ34 (IC 20 nM) and olaparib (IC 5 nM). PJ34 targets mainly PARPs synthesizing proteins but some off-target effects have also been reported, suggesting the effect of PJ34 on cancer cells may not be attributed exclusively to PARP inhibition [22, 23]. For that reason we also used the clinically relevant PARPi olaparib. Following seven days of treatment, neurospheres formation decreased significantly with either PJ34 or olaparib (Figure 1A). Similar results have been reported by Rich and colleagues showing that PARPi preferentially targeted GSCs [16]. Our data support that PARPi targets primary glioma cells in part by perturbing self renewal/GICs phenotype.

PTEN deficiency is one of the most common mutations in human high grade gliomas, and renders these tumours resistant to radio and chemotherapy, conferring increased invasive properties. To further challenge PARPi as anti GBM agents we tested them against established GBM cell lines bearing either wild type or mutant PTEN. Treatment with PARPi of either PTEN wild type or mutant cell lines resulted in loss of cell viability (Figure 1B, figure S1A) and cell death induction (Figure 1C). Due to the previously reported off-target effects of PJ34, the PARP inhibitor olaparib was also tested, exerting similar results (figure S4A). Interestingly, PTEN deficient cells including U87MG displayed an increased sensitivity to PARPi. However, U87MG, which has been previously described to be extremely resistant to apoptotic cell death [24], hardly increased apoptosis following PARPi (Figure 1D, S1B,C) or PARP-1 knockdown (figure S1D) when compared with LN229 (PTEN proficient cell line). Remarkably, PTEN silencing in LN229 cells and PTEN restoration in U87MG cells resulted in increased apoptotic cell death following PARPi (figure S2A,B). This apparently contradictory result may be explained through the genetic context of each cell line: while LN229 cells possess a functional apoptotic machinery that is activated following PARP inhibition, PTEN re-introduction in U87MG cells partially restored apoptotic ability.

Combining PARPi with the methylating agent temozolomide (TMZ) or ionising radiation (IR) did not potentiate cell killing (figure S3A,B,C and data not shown). Thus, PARP inhibition per se was sufficient to induce cell death in PTEN deficient cells more efficiently than the currently used chemotherapeutic drug TMZ or IR. Moreover, the G2/M arrest was also notably diminished in U87MG cells following PARP inhibition respect to PTEN wild type cells (Figures 1E,1F and S4B) and U87MG cells transiently restored with PTEN partially recovered G2/M arrest (figure S2C). In addition, TMZ remarkably induced an arrest in G2/M at 72 hours and the combination with PARPi produced similar effect to PARP inhibition alone (figure S3D).

### PARP inhibition induced down-regulation of the spindle assembly checkpoint protein BUBR1 leading to mitotic instability in PTEN deficient glioma cells

**Figure 2:**
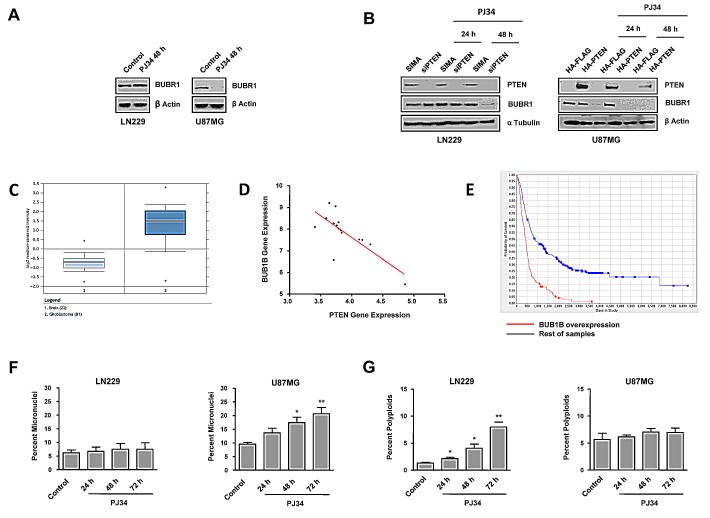
Mitotic instability following PARP inhibition (PJ34 20 μM) A. BUBR1 expression was measured by Western Blot 48 hours after the treatment with PJ34. B. BUBR1 expression was measured by Western Blot after the treatment with PJ34 following PTEN silenting in LN229 cells/PTEN overexpression in U87MG cells. C. BUB1B gene expression analysis obtained with the Oncomine database; p= 2.2E-20, fold change 3.856; number of samples: normal brain n=23, glioblastoma n=81 D. BUB1B gene expression correlates negatively with PTEN expression in GBM low survival patients (less than 12 months); n=15, p<0.001, pearson −0.7592 E. BUB1B overexpression correlates with decreased patient survival. Data obtained from REMBRANDT database. n=413; 138 overexpressing BUB1B and 275 rest of samples. F. Micronuclei formation after DAPI staining was quantified. *p < 0.05, **p < 0.01 *versus* control group by t-test. G. Super G2 fraction, indicating polyploid cells, was analysed by flow cytometry after staining with PI. *p < 0.05, **p < 0.01 *versus* control group by t-test. Data are represented as mean ± SEM of 3 independent experiments.

To further elucidate the mechanistic aspects regarding the effect of PARP inhibition in both PTEN proficient and PTEN mutant GBM cells we explored the induction of genomic instability. PTEN deficient cells lack G2/M arrest following PARPi treatment (Figure 1E and F). The BUBR1 protein ensures accurate segregation of chromosomes through its role in the mitotic checkpoint and the establishment of proper microtubule-kinetochore attachments; and sustained high-level expression of BUBR1 preserves genomic integrity [25]. In Figure 2A we show that PARP inhibition induced BUBR1 down-regulation in U87MG PTEN-deficient cells, suggesting that the Spindle Assembly Checkpoint is compromised in U87MG. Further confirmation for the effect of PARP inhibition on BUBR1 levels was established by the use of a different PARP inhibitor, olaparib, that induced BUBR1 down-regulation in U87MG but not in LN229 cells (figure S4C). Furthermore, silencing PTEN in LN229 cells also results in BUBR1 decrease after PARP inhibition (Figure 2B) while introduction of PTEN in U87MG cells delayed BUBR1 loss (Figure 2B). In addition, in silico analysis using the database Array Express of U87MG cells transduced with wild type PTEN showed a statistically significant decrease in BUB1B expression (the gene for BUBR1) in PTEN transduced cells as well as in the gene coding for the SAC-related factor (and BUBR1 associated protein) AURKB (−2,62 and −3.31 fold decrease respectively). In order to approach the clinical relevance of these variations in BUBR1 levels as function of PTEN we used two available datasets: Oncomine and Array Express from EMBO-EBI. BUB1B gene expression was significantly increased in GBM patients (Figure 2C; p= 2.2E-20, fold change 3.856, number of samples: normal brain n=23, glioblastoma n=81). Moreover, there was an inverse correlation between PTEN and BUB1B expression in GBM patients with low survival (Figure 2D; less than 12 months; n=15, p<0.001, pearson −0.7592) further supporting that targeting BUBR1 (as PARPi does) could be used as rational therapy in PTEN deficient GBM. Interestingly, increased expression of BUB1B correlated with decreased patient survival (Figure 2E).

Another hallmark of genomic instability is micronuclei formation. Following PARP inhibition, U87MG cells, but not LN229 cells displayed a time-dependent accumulation of micronuclei (Figure 2F).

Polyploids are the result of cytokinesis failure after G2/M arrest. However PTEN-deficient cells, unable to activate the G2/M checkpoint, progress to continue cell cycle and complete aberrant mitosis. As shown in Figure 1E, PARP inhibition-induced arrest in G2/M in PTEN-mutant cells was almost suppressed, implying that cells will progress in cell cycle, accumulating genomic instability, and eventually Mitotic Catastrophe but not polyploids (Figure 2G, S4D). Interestingly, and consistent with increased G2/M arrest following PARPi after PTEN restoration (figure S2C), PTEN over-expression in U87MG also increased polyploidy (figure S2D). However, the slight increase observed after long-time of PJ34 treatment suggests a possible interference of the genetic background of each cell line.

Taken together, these results led us to conclude that PARP inhibition compromised mitotic checkpoint through down-regulation of BUBR1, preventing from mitotic arrest only in a PTEN deficient context.

To further understand the impact of PARP inhibition in PTEN mutant cells we performed an expression array focalized in genes involved in cell cycle regulation and DNA repair. In Table 1 we have represented genes whose expression was significantly modified after PARP inhibition in U87MG cells. Up-regulated mRNAs included p53-dependent genes such as BBC3/PUMA (a pro-apoptotic bcl2 and BH3-only pro-apoptotic subclass) and CDKN1A/p21 (pro-apoptotic and CDK2 inhibitor). Up-regulation was also noted in genes involved in DNA damage, G2/M cell cycle checkpoint, and in genes implicated in DNA repair pathways such as XPA, XPC (Nucleotide Excision Repair). A number of down-regulated genes were involved in homologous recombination repair. That is the case for BARD1 and BRIP1, factors associated with BRCA1 who are needed for its activation. Moreover, RAD51 is an essential component of HR repair and its down-regulation could be detrimental for the cell to cope with DNA damage leading to cell death. Chk1, involved in cell cycle arrest after activation of ATM and ATR in response to DNA damage, is also down-regulated as well as the protein phosphatases CDC25A (which is a Chk1 substrate) and CDC25C, involved respectively in G1/S checkpoint and mitosis entry. Gene expression for exonuclease Exo1, that plays a role in mismatch repair, and the endonuclease FEN1, that removes 5′ overhanging flaps in DNA repair and processes the 5′ ends of Okazaki fragments in lagging strand DNA synthesis, is repressed after PARP inhibition. Mutations or deficiency in the Fanconi anemia complementation (FANC) group members is characterized by cytogenetic instability, hypersensitivity to DNA crosslinking agents, increased chromosomal breakage, and defective DNA repair. FANCG gene expression was also down-regulated after PARP inhibition. Phosphorylation of H2AX is involved in the initial early steps of DNA damage response, in the recognition of double strand breaks. Down-regulation of γH2AX is reflecting a strong defective signalling in the initial sensing of DNA lesions. Globally, this perturbation in DNA damage response factors after PARP inhibition suggests a discomposed scenario in the ability of these PTEN-deficient cells to cope with PARP inhibitor-induced DNA lesions that might be therapeutically exploited.

**Table 1:** Expression array of DNA repair proteins in U87MG glioblastoma cells Genes over and underexpressed following 24 hours PJ34 (20 μM) treatment. Data are represented as mean ± SEM of 3 independent experiments. P-value was calculated through t-test.

Genes Over-Expressed	
Gene Symbol	Fold Regulation	P-VALUE
BBC3	2.8101	0.013791
CDKN1A	3.8509	0.000049
XPA	3.3737	0.000464
XPC	2.6833	0.001180

Genes Under-Expressed	
Gene Symbol	Fold Regulation	P-VALUE
BARD1	−3.613	0.043765
BLM	−2.6515	0.040151
BRIP1	−5.1323	0.007620
CDC25A	−3.8447	0.0041 58
CDC25C	−4.9758	0.003132
CHEK1	−2.2562	0.044566
EX01	−4.2833	0.004202
FANCG	−2,1567	0.031524
FEN1	−2.7212	0.030799
H2AFX	−5.4313	0.000279
RAD51	−4.5306	0.001705

### Impaired Homologous Recombination (HR) after PARP inhibition in PTEN deficient glioma cells

**Figure 3:**
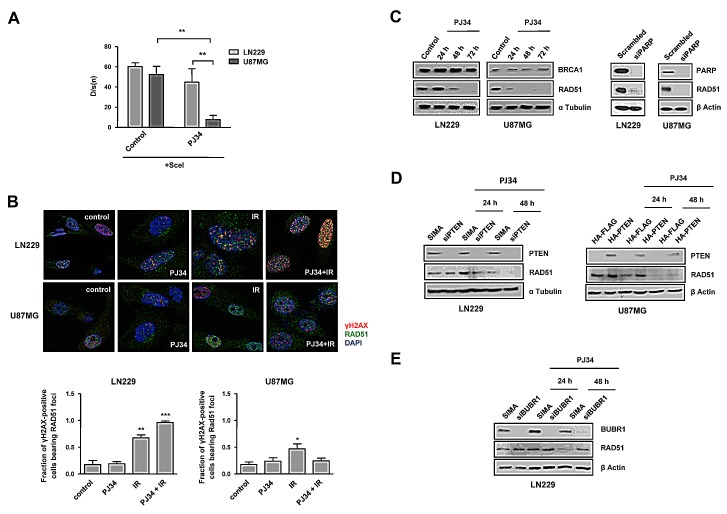
Homologous Recombination (HR) is compromised following PARP inhibition A. Stably transfected with DR-GFP plasmid LN229 and U87MG glioblastoma cell lines were transiently transfected with SceI plasmid. Two days later, they were treated with PARP inhibitor PJ34 (10 μM) for 48 hours. GFP expression was analysed by flow cytometry and the results were processed by Kolmogorov-Smirnov test. **p < 0.01 by t-test. B. Distribution of Rad51 and γH2AX foci; nuclear staining was performed with DAPI. Cells were treated with PJ34 (20 μM) during 48 hours and subsequently irradiated at 2Gy during 4 hours. Fraction of positive γH2AX population bearing Rad51 foci was quantified. *p < 0.05, **p < 0.01, ***p < 0.001 *versus* control group by t-test. C. Homologous-Recombination (HR) proteins BRCA1 and Rad51 expression was analysed by western blot 24, 48 and 72 hours after 20 μM PJ34 treatment and following PARP knockdown. D. RAD51 expression was measured by Western Blot after the treatment with PJ34 following PTEN silenting in LN229 cells/PTEN overexpression in U87MG cells. E. RAD51 expression was measured by Western Blot after the treatment with PJ34 following BUBR1 silenting in LN229 cells. Data are represented as mean ± SEM of 3 independent experiments.

In view of the above results we tested HR efficiency in U87MG and LN229 cell lines containing an integrated copy of the DR-GFP reporter as previously described [26]. This reporter allows to determine the rate of HR repair of a SceI endonuclease-generated DSB in the chromosome by the restoration of an intact green fluorescent protein (GFP) gene. GFP levels were quantified by Kolmogorov-Smirnov adjust, and revealed that LN229 cells expressed higher levels of GFP after transfection, indicating that PTEN mutant cells are compromised in Homologous Recombination, as has been previously reported [20]. Moreover, PARP inhibition further disabled HR, mainly in PTEN mutant cells where we found this repair pathway profoundly down-regulated after the PARPi treatment (Figure 3A).

To confirm the previous results of PARPi inducing increased HR deficiency specifically in PTEN mutant cells, we performed an assay to quantify RAD51 foci, which is also used to assess HR efficiency. First we observed that RAD51 accumulation in U87MG cells did not correlate with the level of DNA damage and did not fluctuate in parallel to γH2AX levels (figure S5A). On the contrary, RAD51 levels in LN229 raised in parallel with γH2AX levels and these foci were resolved following 24 hours after irradiation. These results suggested that PTEN wild type cells, but not PTEN mutant cells, were able to couple HR activation with DNA damage levels. In addition, γH2AX basal levels are much lower in U87MG cells, further confirming the perturbed status of the HR signalling that makes them unable to properly signal and resolve DNA damage (figure S5B).

Consistently, assessing only cell bearing γH2AX foci, we observed that IR-induced accumulation of RAD51 foci was notably reduced in PTEN mutant cells (Figure 3B) and co-treatment with PJ34 further decreased RAD51 foci formation in these cells, supporting the above results obtained with DR-GFP transfection assay. The levels of RAD51 were rapidly down-regulated in U87MG, but not in LN229 where they only decreased after 48 hours of PARP inhibition. PARP-1 silencing, however, affected similarly to RAD51 levels irrespective of the PTEN-status (Figure 3C). Similar results were obtained using the PARPi olaparib (figure S4C). Levels of BRCA1 protein were also reduced in PTEN deficient cells (Figure 3C). To further confirm the association between RAD51 decrease, PARPi treatment and PTEN status, we silenced PTEN in LN229 and we found a decrease in RAD51 levels (Figure 3D); on the other hand, restoring PTEN in U87MG cells led to a delay in RAD51 down-regulation after the treatment (Figure 3D).

As we described above, the expression of the SAC regulatory factor BUBR1 was reduced after PARPi treatment in PTEN mutant cells (Figure 2A). To better understand the association between BUBR1 down-regulation and impaired HR we knocked-down BUBR1 in PTEN proficient cells and we observed a recovery in RAD51 following PARP inhibition (Figure 3E). This apparently paradoxical result might be explained because PARP inhibition, in siBUBR1 cells, is acting in a BUBR1 deficient scenario since the beginning, contrary to the situation on U87MG cells. RAD51 recovery might reflect a compensatory mechanism initiated by the cell to avoid massive DNA damage in the absence of an efficient mitotic checkpoint.

### PARP blockade potentiated *in vitro* and *in vivo* effect of EGFR inhibition on PTEN mutant glioma cells

**Figure 4:**
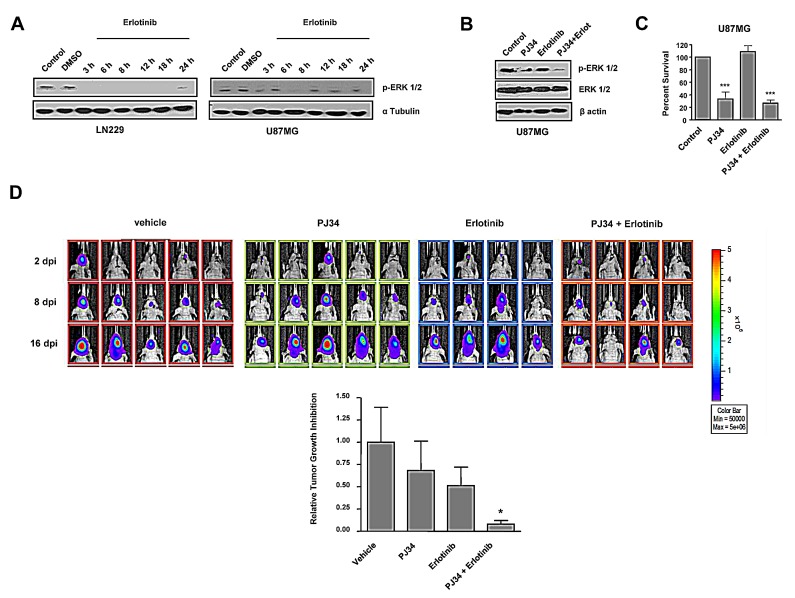
*In vitro* effect of EGFR inhibitor erlotinib and decreased tumours growth *in vivo* after combined treatment with PARP inhibitor and erlotinib A. Western blot analysis of p-ERK-1/2 expression levels at different times following erlotinib treatment.. B,C. B,C. U87MG cells were treated with erlotinib alone or combined with PJ34 during 72 hours. (B) p-ERK-1/2 expression was measured by Western Blot. (C) MTT reduction was analysed. ***p < 0.001 *versus* control group by t-test. D. Mice were inoculated with U87MG-luciferase human cell line. Localization and intensity of luciferase expression were monitored by *in vivo* bioluminiscence imaging (dpi, days post cells injection). Representation of tumours growth inhibition on the 16^th^ day. A statistically significant reduction is observed in the combined treatment of PJ34 and erlotinib. *p < 0.05 *versus* control group by t-test. Data are represented as mean ± SEM of 3 independent experiments.

In spite U87MG glioma cells are not mutant for EGFR, they constitutively activate MAP kinase pathway in virtue of mutations affecting Focal Adhesion Kinases and GRP3 [27, 28]. PARP inhibition did not prevent ERK1/2 activation making this treatment only partially effective in suppressing this proliferative pathway. We reasoned that avoiding signalling arising from EGFR might deregulate the activation of MAP kinase pathway and potentiate the effect of PARP inhibition. While treatment with EGFR inhibitor erlotinib alone prevented ERK1/2 activation in LN229 cells, U87MG cells were refractory to the effect of erlotinib (Figure 4A). Interestingly, co-treatment with PJ34 and erlotinib resulted in a complete suppression of ERK1/2 activation (Figure 4B). Nonetheless, combination of both drugs did not further decrease the PARP inhibitor-induced cytotoxicity (Figure 4C).

The full abrogation of ERK1/2 activation prompted us to test the *in vivo* efficacy of this combination. To this end, we performed an orthotopic assay inoculating U87MG cells that expressed luciferase allowing *in vivo* visualisation of the evolution of tumour mass. While the effect of PJ34 or erlotinib were limited separately (35 and 50% respectively), the combination of both treatments reduced tumour growth to more than 90% after 14 days (Figure 4D). Mice were sacrified after 21 days due excessive tumour growth in vehicle treated mice; at this time PJ34 continued to be effective as anti-tumour agent indicating that the combined inhibition of a pro-survival pathway (using erlotinib) together with the inactivation of HR and the induction of genomic instability by PARP inhibition has a synergic *in vivo* anti-tumour effect.

## DISCUSSION

**Figure 5:**
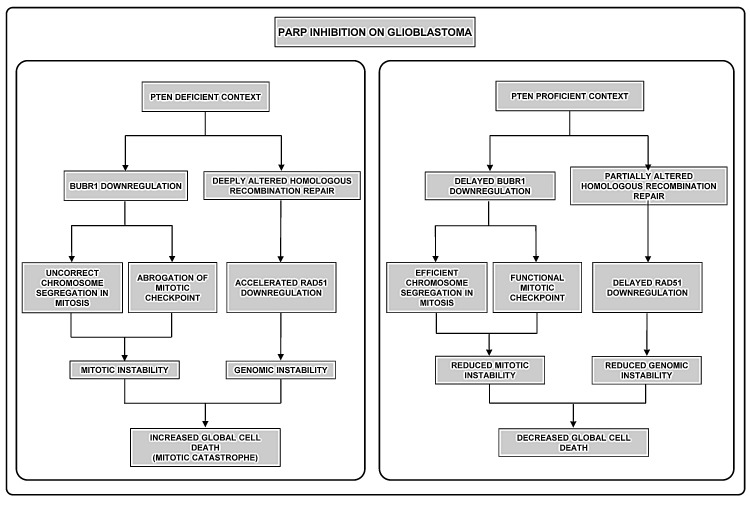
Different effect of PARPi in GBM according to PTEN status First, in the absence of PTEN (left pannel) PARPi perturbs the correct segregation of chromosomes, due to BUBR1 down-regulation; secondly compromised integrity of the genomic material occurs as consequence of the alteration of HR repair. Both situations have been well-described to induce Mitotic Catastrophe. In contrast, in a PTEN-proficient context (right pannel) BUBR1 down-regulation is retarded allowing correct chromosome segregation in mitosis and, secondly, HR repair is less affected. Thus, mitotic and genomic instability are reduced and mitotic catastrophe-independent cell death pathways are activated.

Our reduced understanding of the mechanisms which underlie brain tumorigenesis severely limit preventative and therapeutic options for glioblastoma patients. The current standard treatment for GBM involves surgical resection followed by adjuvant radiotherapy (RT) with ionising radiation, with or without concomitant chemotherapy. Disappointingly, this regimen only affords GBM patients a median survival benefit of 14.6 months- a 12 month improvement over resection alone. It is important to note that radio- and chemotherapies are, at the molecular level, based on inducing enough DNA damage in the tumour cell to result in lethality. The results shown in this study reflect that PARP inhibition results in a profound alteration of genomic instability in PTEN-deficient glioma cells affecting cell cycle, mitotic checkpoint and homologous recombination, leading to micronuclei formation, as hallmark of MC (Figure 5). The protein kinase BUBR1 plays a key role in the maintenance of chromosomal stability [29] owing to its involvement in the formation of proper chromosome-spindle attachments. First, BUBR1 down-regulation activates Aurora B kinase and the kinetochore protein CENP-A, resulting in the loss of kinetochore-microtubule attachments [30]. Second, BUBR1 avoids sister chromatids separation in anaphase when chromosome-spindle attachments are uncorrect, since it is involved on the Mitotic Checkpoint (Spindle Assembly Checkpoint or SAC) [31]. When interactions between kinetochores and microtubules are unstable in metaphase, BUBR1, acting together with MAD2 and BUB3, join CDC20 avoiding APC activation thus preventing sister chromatids separation in anaphase. Remarkably gene expression of BUB1B (the gene coding for BUBR1) is very significantly up-regulated in GBM patients and its expression was inversely correlated with PTEN in short-survival patients. In line with this finding recently it has been described that BUB1B is differentially required for GSCs expansion in glioblastoma tumours and genetically transformed cells that have added requirement for BUB1B to suppress lethal consequences of altered kinetochore [32].

In the current study we have found that Homologous Recombination Repair deficiency in PTEN mutant glioma cells is further disabled after PARP inhibition due in part to RAD51 down-regulation. A previous study has shown that PARPi down-regulates RAD51 and BRCA1 leading to HR deficiency at times where an elevated loss of cell viability was observed [33]. In our study, PARP inhibition leads to a profound amplification of HR deficiency in PTEN mutant cells already at 24 hours (Figure 3C and Table 1), where no cytotoxic effect is still appreciated (Figure 1A). Moreover, the expression pattern of key components of the DNA damage response is strongly affected after blunting PARP activity, (PARP-1 of other PARP family members with poly (ADP-ribosyl)ation activity), including perturbation of the HR machinery and other DNA repair pathways as well as factors involved in genomic instability, beyond affecting RAD51. PTEN restoration in U87MG slowed RAD51 decrease, whereas PTEN silencing in LN229 augmented RAD51 down-regulation after PARP inhibition. Thus, we show that PTEN is involved in the mechanism by which PARP inhibition is disabling Homologous Recombination machinery, which should be taken into account in a possible clinical setting.

The benefit of combining PARP inhibitors with currently used chemotherapy has been largely reported including the potentiating effect of PJ34 [34, 35]. Here we also show compelling evidences that cell death pathway by which PARP inhibition impacts on cell viability of PTEN-deficient cells is MC. MC is a mechanism activated following genomic instability. It senses mitotic failure and responds to it by driving the cell to an irreversible fate, be it apoptosis, necrosis or senescence [36, 37]. The stimuli and perturbations that are described to trigger MC can be divided in two groups. The first group of inducers interfere with the faithful segregation of chromosomes in mitosis. The second group directly affects the integrity of the genetic material, for example DNA-damaging agents or compromised DNA-repair pathways. Thus, in our case, aberrant mitotic spindle organization and DNA segregation due to BUBR1 down-regulation constitute a “first-group inducer” of MC while impaired HR repair due to compromised RAD51 is “second trigger” of MC. However, in spite of the increasingly detailed description of the mechanisms that precede and follow MC, the molecular bridges between mitotic aberrations and cell death are still largely elusive.

In an attempt to increase the *in vivo* cell killing effect of PARP inhibition on glioma cells we ideated (given the up-regulation of pro-survival signalling pathways in PTEN-deficient glioma cells) the co-treatment with an inhibitor of EGFR to disable pro-survival signals. Although no *in vitro* potentiation by erlotinib of PARP-induced cytotoxic effect was observed, this combination was very effective in the suppression of ERK1/2 activation (Figure 4B) and, more interestingly, *in vivo* co-treatment was synergic in slowing-down tumour growth. This enhanced *in vivo* potentiation using co-treatment of anti-neoplastic agents with PARP inhibitors has been already described for different preclinical models and has been related to the increased vascular function after inhibition of PARP resulting in amelioration of drug availability in the tumour milieu [38]. Moreover, besides this general property of PARP inhibitors, the use of these compounds takes advantage of the elevated propensity to display genomic instability (which is related with aggressiveness trait) of PTEN deficient glioma cells. The ultimate mechanism underlying the synergistic effect of PARP1 and EGFR remains to be elucidated, but one possibility is that in specific settings inhibition of PARP with PJ34 activates pro-survival pathways as has been shown for p38/MAPK during osteoclast differentiation [39]. A different study has reported that PARP inhibitors may target different kinases in a cell-specific manner [40]. In addition to the shut-down of EGFR signalling, other combinatorial treatments could be envisaged based on the rational knowledge of glioma cells molecular alterations. Another broad field to explore is the use of PARP inhibitors to act as radio-potentiators against GBM and overcome tumour resistance to standard radiation therapy. In summary PARP inhibitors represent an exciting new class of antineoplasic drugs and there may well have much wider clinical indications not just restricted to BRCA1/2 mutant tumours but to others where PARP inhibitor treatment enhance HR deficiency and mitotic alterations, driving the cell towards a status of genomic instability.

## MATERIALS AND METHODS

### Cell Culture and Treatments

U87MG and U118MG PTEN mutant and LN229 PTEN wild type (wt) glioblastoma cell lines as well as SW1783 PTEN mutant grade III astrocytoma cell line, were cultured in Dulbeco's Modified Eagle's Medium (DMEM) supplemented with 10 % inactive fetal bovine serum (FBSi, Gibco Invitrogen), at 37 ºC in a humidified 5% CO_2_ atmosphere.

Patient-derived Glioma Stem Cell TG1 was obtained as described previously [41] and maintained in DMEM/F12 plus N2, G5 and B27 (Invitrogen) at 37 ºC in a humidified 5% CO_2_ atmosphere.

PARP inhibitors PJ34 (Alexis Biochemicals) and AZD2281/olaparib (Deltaclon) were used. Olaparib was dissolved in DMSO and PJ34 was dissolved in water. Both were stored at −20ºC. Cells were treated with 10 μM olaparib or 10 to 20 μM PJ34 during 24, 48 or 72 hours.

Temozolomide (T2577-25MG Sigma-Aldrich, St Louis, USA) was dissolved in DMSO and stored at −20ºC. Cells were treated with 100 μM Temozolomide during 24, 48 or 72 hours.

### Viability Assays

Viability decrease was determined using MTT and Propidium Iodide. For MTT (3-(4, 5-Dimethylthiazol-2-yl)-2,5-diphenyl Tetrazolium Bromide) assay, cells were plated in 96 wells at a density of 8 × 10^3^ cells. MTT assay was performed using Cell Proliferation Kit I (MTT, 1-65-007, Roche, Mh Germany) following manufacturer's instructions. Global cell death assay was performed with Trypan Blue (Fluka analytical, 93595). Cells were plated in 24 wells at a density of 2 × 10^4^ cells. One week after the treatment cells were trypsinized, washed with PBS and stained with Trypan Blue. Finally, cells were placed in a Neubauer counting chamber and counted in order to check the rate of blue cells (indicating dead cells) in the population.

### Neurosphere Formation Assay

GSCs were dissociated by up-and-down pipetting and plated in 48 wells. PARP inhibitor was added every 24 hours during the three first days of experiment [41]. GSCs were dissociated every day in order to check their ability to form seconday neurospheres at the end of the experiment. The 7^th^ day, counts were blindly performed on 10 fields of view, and the mean number of neurospheres per field of view was calculated.

### Cell Cycle Assay

Cell cycle was analysed by flow cytometry [42]. Cells were plated in 6 wells at a density of 1.5 × 10^5^ cells. After the treatments, cells were trypsinized, washed with PBS, permeabilized with 70% ice cold ethanol, washed again with PBS and incubated with propidium iodide and 100 μg/ml RNAase A (Ribonuclease A from bovine pancreas R6513-10MG Sigma-Aldrich, St Louis, USA) for 20 min. When GFP population was examined, cell fixation before permeabilization was required, as previously described by Lamm et al [42]. Cells were analysed on a FACScan using CellQuest software, and cell cycle was determined using FlowJo software. In the case of PTEN overexpression, GFP population was gated in order to analyze cell cycle in a 100% transfected population.

### Apoptosis Assays

Apoptosis was determined by different methods. a) Cell Cycle Assay: Sub G1 population was determined through Cell Cycle assay, as described above. b) Pyknotic nuclei: Cells were plated in 6 wells at a density of 5 × 10^4^ cells per well. 72 hours after the treatments cells were fixed in Paraformaldehyde (4%, wt/vol in PBS1x with 2% Sucrose) for 10 minutes at room temperature and incubated with DAPI for 10 minutes. The number of cells with nuclear apoptotic morphology was determined using a Zeiss Fluorescence Microscope. c) Caspase 3/7 activity: Cells were plated in 96 wells at a density of 6 × 10^3^ cells per well. Following the treatments, The Caspase - Glo reagent (Promega) was added directly to cells and incubated at room temperature for 30 minutes before recording luminiscence in a TECAN infinite 200 Luminometer. Each point represents the average of 3 wells per condition to 3 independent experiments.

### Western Blot Analysis

Cells were plated in 6 wells at a density of 2 × 10^5^ cells per well. After PARP inhibition, cells were washed twice with PBS and resuspended in 200 μl of TR3 Lysis Buffer (3% SDS, 10% Glycerol, 10mM Na_2_HPO_4_ anhidro). Then cells were sonicated and 20 μl of 50% β-mercaptoethanol - 50% Bromofenol blue were added. The protein concentration was determined using the Lowry assay. Proteins were resolved on SDS-polyacrylamide gels and transferred onto PVDF Membrane (Biorad). The blot was blocked with 5% milk powder in PBS1X with 0.1% Tween-20 for 60 minutes and incubated overnight with 1% milk powder in PBS1X with 0.1% Tween-20 with the following antibodies: anti-PARP-1(C2-10 mouse, ALEXIS, LA), anti-PTEN (Santa Cruz Biotechnology sc-7974), anti-BRCA1 (Santa Cruz Biotechnology sc-642), anti-RAD51 (Santa Cruz Biotechnology (H-92) sc-8349), anti-phosphoERK (Santa Cruz Biotechnology sc-7383), anti-ERK (Invitrogen. Carlsbad, CA 61-7400), anti-phospho H2AX (Millipore 05-636) and anti-BUBR1 (BD Bioscience. Erembodegem, Belgium). α-Tubulin (Sigma, St Louis MO) and β-Actin (Sigma, St Louis MO) were used as loading control. Bands were visualized with ECL, ECL-PLUS and ECL PRIME (Amersham Biosciences) and the pictures were taken with the imaging system ChemiDoc XRS System (BIO-RAD) and medical X-ray films (AGFA).

### RNA interference

Cells were plated in 6 wells at a density of 9 × 10^4^ cells per well. 24 hours later, cells were transfected with the indicated siRNAs at 50 nM using Lipofectamine 2000 transfection agent (Invitrogen) according to the manufacturer's guide. Double-stranded RNA duplexes corresponding to a non-targeted control (5′–CCUACAUCCCGAUCGAUGAUGUU-3′), PTEN (5′-GCUACCUGUUAAAGAAUCA-3′) and BUBR1 (5′-CGGGCAUUUGAAUAUGAAA-3′) were ordered to SIGMA-ALDRICH, and double-stranded RNA duplexes corresponding to human PARP-1 were from Ambion Applied Biosystems. 48 hours after transfection, cells were treated with PARP inhibitors as indicated above.

### PTEN restoration

Cells were plated in 6 wells at a density of 1 × 10^5^ cells per well. 24 hours later, transfection was performed with 0,5 μg pSG5L Flag HA Plasmid, pSG5L Myr HA PTEN Plasmid, GFP-PTEN Plasmid or pCDNA3-GFP Plasmid (all from Addgene), using JetPRIME TM (Polyplus transfection, Illkirch, France) according to the manufacturer's protocol. Cells were treated 48 hours after the transfection and harvested following the treatment in order to develop Cell Cycle and Western Blot analysis.

### Immunofluorescence

Cells were plated in 12 wells at a density of 2,5 × 10^4^ cells per well on glass cover-slips. After the treatments cells were fixed with Paraformaldehyde Solution (4%, wt/vol in PBS1x with 2% Sucrose) for 10 minutes at room temperature and permeabilized with PBS 1X 0,5% Triton x100 for 5 minutes at room temperature. phospho-H2AX was detected by immunofluorescence, using monoclonal antibody (Millipore 05-636) at a dilution 1:250 and FITC-conjugated goat anti-mouse immunoglobulin (Sigma, St Louis Mo) at a dilution 1:400. RAD51 foci were detected using rabbit polyclonal IgG antibody (H-92 Santa Cruz) 1:100 and FITC-conjugated goat anti-rabbit 1:400. Nuclear counterstaining with DAPI was performed after removal of excess secondary antibody. Immunostaining was visualized with Confocal Leica LCS SP5 Fluorescence Microscope.

### Homologous Recombination Assay

U87MG and LN229 glioma cells were stably transfected with a pDR-GFP plasmid containing a mutated GFP gene with an 18 bp SceI site and were maintained under puromycin selection before use. Transient transfection of SceI in both cell lines creates a DSB at the relevant site in the integrated GFP gene. Homologous recombination repair (HRR) of this break restores GFP gene expression [26].

Cells were plated in 6 wells at a density of 9 × 10^4^ cells per well for stable transfection with DR-GFP. 24 hours later, cells were transfected with 1 μg DR-GFP plasmid per well using JetPEI TM (Polyplus transfection, Illkirch, France), according to the manufacturer's protocol. Transfected cells were maintained under puromycin selection, and transfection was proved by PCR with the primers

DRGFP1 5′AGGGCGGGGTTCGGCTTCTGG 3′

DRGFP2 5′CCTTCGGGCATGGCGGACTTGA 3′

For the transient transfection with SceI, cells were plated in 6 wells at a density of 9 × 10^4^ cells per well. 24 hours later, cells were transfected with 4 μg SceI plasmid per well using JetPRIME^TM^ (Polyplus transfection, Illkirch, France) according to the manufacturer's protocol. 24 hours after the transfection, cells were treated with PJ34 hours during 48 hours. Finally, cells were trypsinized and percentage of GFP expressing cells was measured by flow cytometry on a FACScan.

Frequency of recombination events was calculated as mean percentage of GFP positive cells transfected with SceI divided by mean percentage of GFP positive cells transfected with pEGFP. Results were represented through Kolmogorov-Smirnov adjust using CellQuest software.

Additionally, homologous recombination was indirectly evaluated by the number of cells with rad51 foci to get the fraction of γH2AX positive cells bearing Rad51 foci. Alt least 120 cells from three independent experiments were counted using Image J software.

### Micronucleus assay

DAPI counterstain described for pyknotic nuclei quantification was also used in order to analyse micronuclei frequency after PARP inhibition. Micronuclei counting was performed using Image J software.

### DNA repair microarray

Cells were plated in p60 at a density of 1 × 10^6^ cells. RNA extraction was performed 24 hours after PJ34 treatment, with RNeasy MiniKit (Qiagen). Retrotranscription was developed with RT2 First Strand kit (Qiagen) and cDNA was tested by RT2 Profiler PCR Array - Human DNA Damage Signalling Pathway (Qiagen) according to the manufacturer's protocol. Data were analysed by the ΔΔCt method.

### Ethics Statement

All human subjects data was publicly available in de-identified form on the Oncomine. website (https://www.oncomine.org/) Therefore, its use was not classified as human subjects research, and no Institutional Review Board approval was needed.

### Patient Datasets and Data Analysis

The microarray gene expression data was obtained from EMBO-EBI (http://www.ebi.ac.uk/arrayexpress/) and the clinical data was obtained from the database Oncomine (https://www.oncomine.org/) using data available on October 1st, 2010. Diagnoses were also made at the respective clinics. At the time of access, 343 glioma patient samples with both gene expression data and corresponding survival times were available on the Rembrandt database. These included 413 GBMs, 138 overexpressing BUB1B and 275 rest of samples.

### *In vivo* bioluminescence assay

This study was performed in strict accordance with the recommendations in the Guide for the Care and Use of Laboratory Animals of the Bioethical Committee of CIBM-UGR. The protocol was approved by the Committee on the Ethics of Animal Experiments of the CIBM-UGR. All surgery was performed under ketamine – xylazine anesthesia, and every effort was made to minimize suffering.

Thirteen-weeks-old male Balb/cnu/nu mice (Charles River Laboratories, Wilmington, MA, USA) were injected intracraneally with U87MG-luc cells (1 × 10^5^) by introducing stereotactically the needle of a Hamilton syringe. The day after injection of tumour cells mice were treated three times per week mice with PJ34 at a dose of 10mg/kg body weight and/or erlotinib at a dose of 50 mg/kg body weight injected intraperitoneally. Sodium Chloride solution/60% DMSO was used as vehicle. In order to develop *in vivo* bioluminiscence measurement, mice were injected intraperitoneally with D-luciferin solution dissolved in phosphate-buffered saline at a dose of 150 mg/kg body weight. After 5 minutes, the animals were anesthetized in the dark chamber using 3% isoflurane in air at 1.5 L/min and O2 at 0.2 L/min/mouse, and animals were imaged in a chamber connected to a camera (IVIS, Xenogen, Alameda, CA). The quantification of light emission was performed in photons/second/cm^2^/steradian using Living Image 2.6.1 software (Xenogen). Tumour growth was monitored at 0, 2, 8, 15 and 21 days by *in vivo* imaging and bioluminiscence measurement. After 21 days, mice were sacrificed, and brains were dissected and placed in Petri dishes with D-luciferin solution at a dose of 20μg/ml. Ex vivo quantification of light emission was performed by introducing the petri dishes inside the chamber connected to IVIS as explained before.

### Statistical Analysis

Independent experiments were pooled when the coefficient of variance could be assumed identical. Statistical significance was evaluated using t-test (n=number of independent experiments). *P*-values below 0.05 were considered significant.

## SUPPLEMENTARY MATERIAL, FIGURES



## References

[1] Furnari FB, Fenton T, Bachoo RM, Mukasa A, Stommel JM, Stegh A, Hahn WC, Ligon KL, Louis DN, Brennan C, Chin L, DePinho RA, Cavenee WK (2007). Malignant astrocytic glioma: genetics, biology, and paths to treatment. Genes & development.

[2] Comprehensive genomic characterization defines human glioblastoma genes and core pathways (2008). Nature.

[3] Parsons DW, Jones S, Zhang X, Lin JC, Leary RJ, Angenendt P, Mankoo P, Carter H, Siu IM, Gallia GL, Olivi A, McLendon R, Rasheed BA, Keir S, Nikolskaya T, Nikolsky Y (2008). An integrated genomic analysis of human glioblastoma multiforme. Science.

[4] Salmena L, Carracedo A, Pandolfi PP (2008). Tenets of PTEN tumor suppression. Cell.

[5] Shen WH, Balajee AS, Wang J, Wu H, Eng C, Pandolfi PP, Yin Y (2007). Essential role for nuclear PTEN in maintaining chromosomal integrity. Cell.

[6] Schreiber V, Dantzer F, Ame JC, de Murcia G (2006). Poly(ADP-ribose): novel functions for an old molecule. Nat Rev Mol Cell Biol.

[7] Gibson BA, Kraus WL (2012). New insights into the molecular and cellular functions of poly(ADP-ribose) and PARPs. Nat Rev Mol Cell Biol.

[8] Bryant HE, Schultz N, Thomas HD, Parker KM, Flower D, Lopez E, Kyle S, Meuth M, Curtin NJ, Helleday T (2005). Specific killing of BRCA2-deficient tumours with inhibitors of poly(ADP-ribose) polymerase. Nature.

[9] Farmer H, McCabe N, Lord CJ, Tutt AN, Johnson DA, Richardson TB, Santarosa M, Dillon KJ, Hickson I, Knights C, Martin NM, Jackson SP, Smith GC, Ashworth A (2005). Targeting the DNA repair defect in BRCA mutant cells as a therapeutic strategy. Nature.

[10] McCabe N, Turner NC, Lord CJ, Kluzek K, Bialkowska A, Swift S, Giavara S, O'Connor MJ, Tutt AN, Zdzienicka MZ, Smith GC, Ashworth A (2006). Deficiency in the repair of DNA damage by homologous recombination and sensitivity to poly(ADP-ribose) polymerase inhibition. Cancer Res.

[11] Fong PC, Boss DS, Yap TA, Tutt A, Wu P, Mergui-Roelvink M, Mortimer P, Swaisland H, Lau A, O'Connor MJ, Ashworth A, Carmichael J, Kaye SB, Schellens JH, de Bono JS (2009). Inhibition of poly(ADP-ribose) polymerase in tumors from BRCA mutation carriers. N Engl J Med.

[12] Sun CK, Zhang F, Xiang T, Chen Q, Pandita TK, Huang Y, Hu MC, Yang Q (2014). Phosphorylation of ribosomal protein S6 confers PARP inhibitor resistance in BRCA1-deficient cancers. Oncotarget.

[13] Ha K, Fiskus W, Choi DS, Bhaskara S, Cerchietti L, Devaraj SG, Shah B, Sharma S, Chang JC, Melnick AM, Hiebert S, Bhalla KN (2014). Histone deacetylase inhibitor treatment induces ‘BRCAness’ and synergistic lethality with PARP inhibitor and cisplatin against human triple negative breast cancer cells. Oncotarget.

[14] Ossovskaya V, Koo IC, Kaldjian EP, Alvares C, Sherman BM (2010). Upregulation of Poly (ADP-Ribose) Polymerase-1 (PARP1) in Triple-Negative Breast Cancer and Other Primary Human Tumor Types. Genes Cancer.

[15] Murai J, Zhang Y, Morris J, Ji J, Takeda S, Doroshow JH, Pommier Y (2014). Rationale for poly(ADP-ribose) polymerase (PARP) inhibitors in combination therapy with camptothecins or temozolomide based on PARP trapping versus catalytic inhibition. J Pharmacol Exp Ther.

[16] Venere M, Hamerlik P, Wu Q, Rasmussen RD, Song LA, Vasanji A, Tenley N, Flavahan WA, Hjelmeland AB, Bartek J, Rich JN (2014). Therapeutic targeting of constitutive PARP activation compromises stem cell phenotype and survival of glioblastoma-initiating cells. Cell Death Differ.

[17] van Vuurden DG, Hulleman E, Meijer OL, Wedekind LE, Kool M, Witt H, Vandertop PW, Wurdinger T, Noske DP, Kaspers GJ, Cloos J (2011). PARP inhibition sensitizes childhood high grade glioma, medulloblastoma and ependymoma to radiation. Oncotarget.

[18] McEllin B, Camacho CV, Mukherjee B, Hahm B, Tomimatsu N, Bachoo RM, Burma S PTEN loss compromises homologous recombination repair in astrocytes: implications for glioblastoma therapy with temozolomide or poly(ADP-ribose) polymerase inhibitors. Cancer research.

[19] Mendes-Pereira AM, Martin SA, Brough R, McCarthy A, Taylor JR, Kim JS, Waldman T, Lord CJ, Ashworth A (2009). Synthetic lethal targeting of PTEN mutant cells with PARP inhibitors. EMBO Mol Med.

[20] McEllin B, Camacho CV, Mukherjee B, Hahm B, Tomimatsu N, Bachoo RM, Burma S (2010). PTEN loss compromises homologous recombination repair in astrocytes: implications for glioblastoma therapy with temozolomide or poly(ADP-ribose) polymerase inhibitors. Cancer research.

[21] Galan-Moya EM, Le Guelte A, Lima Fernandes E, Thirant C, Dwyer J, Bidere N, Couraud PO, Scott MG, Junier MP, Chneiweiss H, Gavard J (2011). Secreted factors from brain endothelial cells maintain glioblastoma stem-like cell expansion through the mTOR pathway. EMBO reports.

[22] Castiel A, Visochek L, Mittelman L, Zilberstein Y, Dantzer F, Izraeli S and, Cohen-Armon M (2013). Cell death associated with abnormal mitosis observed by confocal imaging in live cancer cells. J Vis Exp.

[23] Castiel A, Visochek L, Mittelman L, Dantzer F, Izraeli S, Cohen-Armon M (2011). A phenanthrene derived PARP inhibitor is an extra-centrosomes de-clustering agent exclusively eradicating human cancer cells. BMC Cancer.

[24] Sgorbissa A, Tomasella A, Potu H, Manini I, Brancolini C (2011). Type I IFNs signaling and apoptosis resistance in glioblastoma cells. Apoptosis : an international journal on programmed cell death.

[25] Baker DJ, Dawlaty MM, Wijshake T, Jeganathan KB, Malureanu L, van Ree JH, Crespo-Diaz R, Reyes S, Seaburg L, Shapiro V, Behfar A, Terzic A, van de Sluis B, van Deursen JM (2013). Increased expression of BubR1 protects against aneuploidy and cancer and extends healthy lifespan. Nature cell biology.

[26] Weinstock DM, Nakanishi K, Helgadottir HR, Jasin M (2006). Assaying double-strand break repair pathway choice in mammalian cells using a targeted endonuclease or the RAG recombinase. Methods Enzymol.

[27] Park MJ, Kim MS, Park IC, Kang HS, Yoo H, Park SH, Rhee CH, Hong SI, Lee SH (2002). PTEN suppresses hyaluronic acid-induced matrix metalloproteinase-9 expression in U87MG glioblastoma cells through focal adhesion kinase dephosphorylation. Cancer research.

[28] Clark MJ, Homer N, O'Connor BD, Chen Z, Eskin A, Lee H, Merriman B, Nelson SF (2010). U87MG decoded: the genomic sequence of a cytogenetically aberrant human cancer cell line. PLoS genetics.

[29] Ricke RM, van Deursen JM (2013). Aneuploidy in health, disease, and aging. J Cell Biol.

[30] Lampson MA, Kapoor TM (2005). The human mitotic checkpoint protein BubR1 regulates chromosome-spindle attachments. Nat Cell Biol.

[31] Musacchio A, Salmon ED (2007). The spindle-assembly checkpoint in space and time. Nat Rev Mol Cell Biol.

[32] Ding Y, Hubert CG, Herman J, Corrin P, Toledo CM, Skutt-Kakaria K, Vazquez J, Basom R, Zhang B, Risler JK, Pollard SM, Nam DH, Delrow JJ, Zhu J, Lee J, DeLuca J (2013). Cancer-Specific requirement for BUB1B/BUBR1 in human brain tumor isolates and genetically transformed cells. Cancer Discov.

[33] Hegan DC, Lu Y, Stachelek GC, Crosby ME, Bindra RS, Glazer PM (2010). Inhibition of poly(ADP-ribose) polymerase down-regulates BRCA1 and RAD51 in a pathway mediated by E2F4 and p130. Proc Natl Acad Sci U S A.

[34] Tentori L, Ricci-Vitiani L, Muzi A, Ciccarone F, Pelacchi F, Calabrese R, Runci D, Pallini R, Caiafa P, Graziani G (2014). Pharmacological inhibition of poly(ADP-ribose) polymerase-1 modulates resistance of human glioblastoma stem cells to temozolomide. BMC Cancer.

[35] Tang JB, Svilar D, Trivedi RN, Wang XH, Goellner EM, Moore B, Hamilton RL, Banze LA, Brown AR, Sobol RW (2011). N-methylpurine DNA glycosylase and DNA polymerase beta modulate BER inhibitor potentiation of glioma cells to temozolomide. Neuro Oncol.

[36] Galluzzi L, Vitale I, Abrams JM, Alnemri ES, Baehrecke EH, Blagosklonny MV, Dawson TM, Dawson VL, El-Deiry WS, Fulda S, Gottlieb E, Green DR, Hengartner MO, Kepp O, Knight RA, Kumar S (2012). Molecular definitions of cell death subroutines: recommendations of the Nomenclature Committee on Cell Death 2012. Cell Death Differ.

[37] Vitale I, Galluzzi L, Castedo M, Kroemer G (2011). Mitotic catastrophe: a mechanism for avoiding genomic instability. Nature reviews Molecular cell biology.

[38] Ali M, Telfer BA, McCrudden C, O'Rourke M, Thomas HD, Kamjoo M, Kyle S, Robson T, Shaw C, Hirst DG, Curtin NJ and, Williams KJ (2009). Vasoactivity of AG014699, a clinically active small molecule inhibitor of poly(ADP-ribose) polymerase: a contributory factor to chemopotentiation *in vivo*?. Clin Cancer Res.

[39] Robaszkiewicz A, Valko Z, Kovacs K, Hegedus C, Bakondi E, Bai P, Virag L (2014). The role of p38 signaling and poly(ADP-ribosyl)ation-induced metabolic collapse in the osteogenic differentiation-coupled cell death pathway. Free Radic Biol Med.

[40] Antolin AA, Mestres J (2014). Linking off-target kinase pharmacology to the differential cellular effects observed among PARP inhibitors. Oncotarget.

[41] Patru C, Romao L, Varlet P, Coulombel L, Raponi E, Cadusseau J, Renault-Mihara F, Thirant C, Leonard N, Berhneim A, Mihalescu-Maingot M, Haiech J, Bieche I, Moura-Neto V, Daumas-Duport C, Junier MP (2010). CD133, CD15/SSEA-1, CD34 or side populations do not resume tumor-initiating properties of long-term cultured cancer stem cells from human malignant glio-neuronal tumors. BMC Cancer.

[42] Lamm GM, Steinlein P, Cotten M, Christofori G (1997). A rapid, quantitative and inexpensive method for detecting apoptosis by flow cytometry in transiently transfected cells. Nucleic Acids Res.

