# Supplementary material for: PARP targeting counteracts gliomagenesis through induction of mitotic catastrophe and aggravation of deficiency in homologous recombination in PTEN-mutant glioma
Source: Oncotarget. 2014 Dec 11;6(7):4790–803. doi: 10.18632/oncotarget.2993 (PMC4467115; doi:10.18632/oncotarget.2993)
Supplement: Supplementary file 1 [file oncotarget-06-4790-s001.pdf]

PARP targeting counteracts gliomagenesis through induction of mitotic catastrophe and aggravation of deficiency in homologous recombination in PTEN-mutant glioma

Supplementary Material

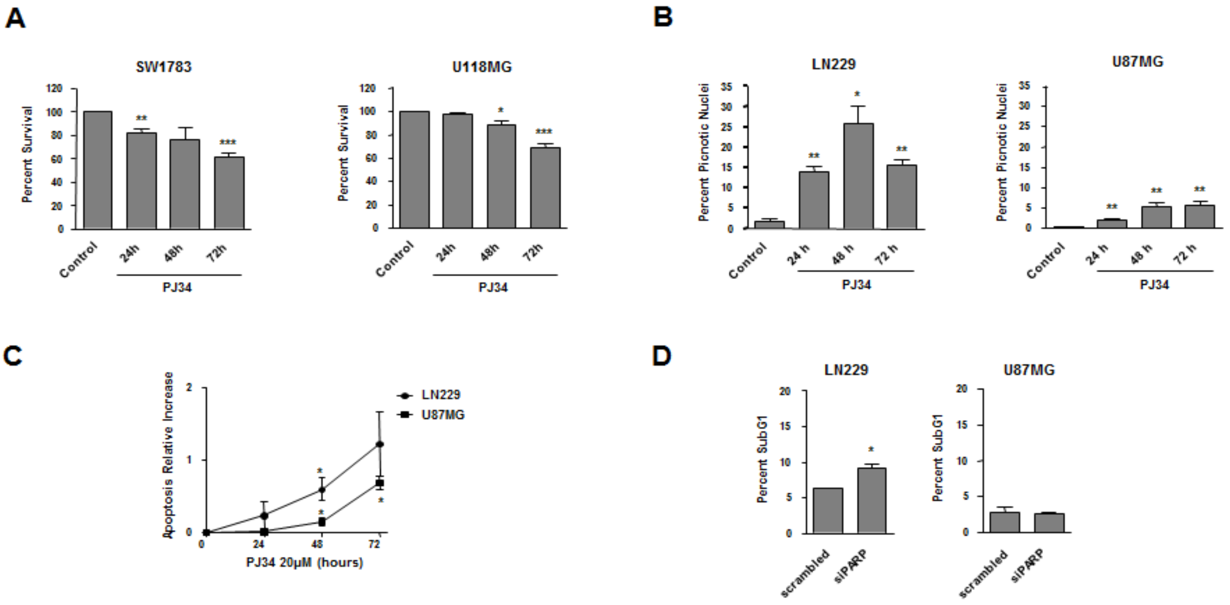

Figure S1: Cell viability in different GBM cell lines after treatment with PARP inhibitor PJ34 (20 μM) or PARP-1 knockdown. Effect of PTEN on cell viability.

A. Viability analysis by MTT assay of PTEN mutant glioblastoma cells SW1783 and U118MG treated for 24, 48 and 72 hours. Data were normalized and expressed as a percentage of the control. *$p < 0.05$, **$p < 0.01$, ***$p < 0.001$ by t-test.

B,C. Apoptosis activation was determined 24, 48 and 72 hours after the treatment. **(B)** Picnotic nuclei counting (ten fields of view per condition) was performed after the treatments. **(C)** Caspase 3 activation was measured. Data were taken as Relative Luminescence Units (RLU), normalized and expressed as a fraction of the control. *$p < 0.05$, **$p < 0.01$, *versus* control group by t-test.

D. PARP-1 was silenced in both LN229 and U87MG cell lines. SubG1 fraction was analysed by flow cytometry following staining with PI. *$p < 0.05$ *versus* control group by t-test.

Data are represented as mean ± SEM of 3 independent experiments.

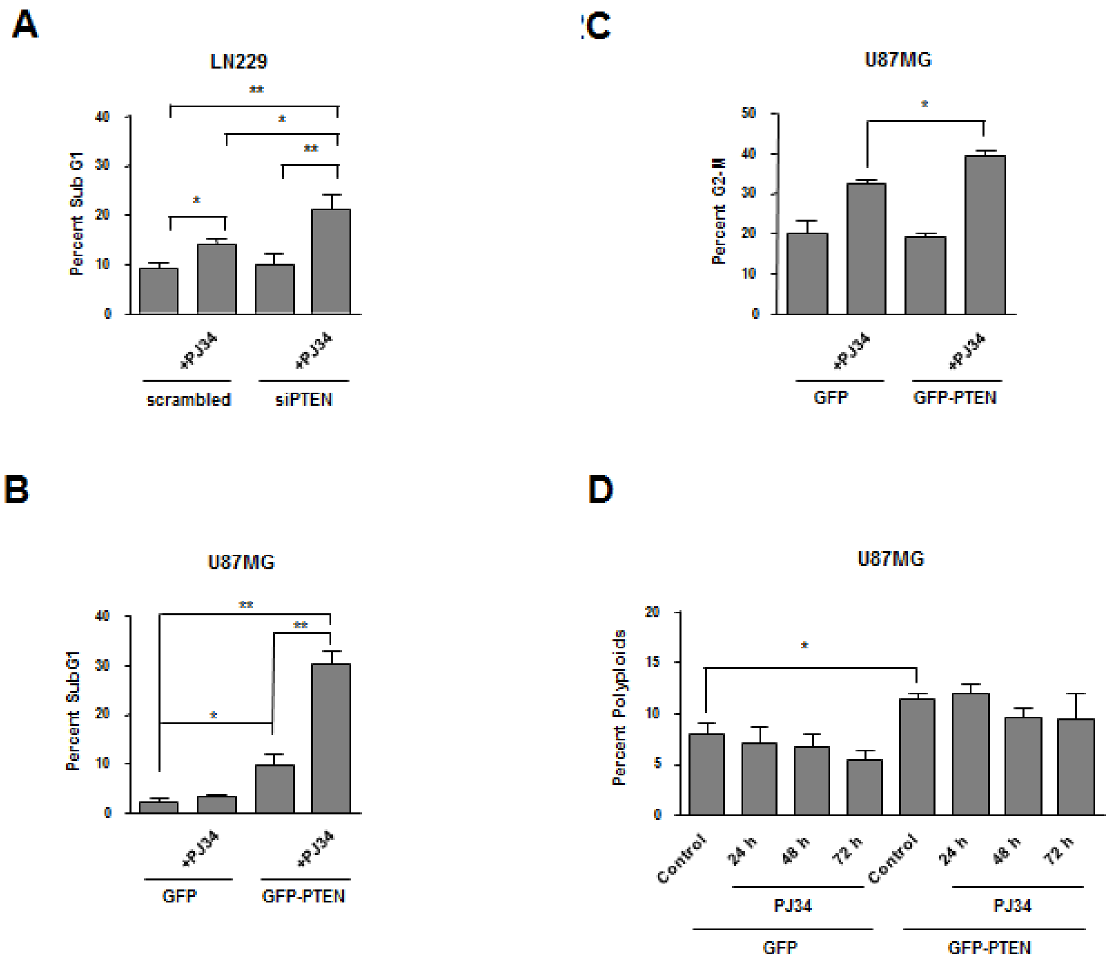

Figure S2: Effect of PTEN silencing/PTEN restoration on cell cycle.

A. PTEN was silenced in LN229 PTEN proficient cells and SubG1 fraction was analysed 72 hours after the treatment. *$p < 0.05$, **$p < 0.01$ *versus* control group by t-test.

B. PTEN-GFP was overexpressed in U87MG PTEN mut cells and SubG1 fraction of GFP-positive cells was analysed 72 hours after the treatment. *$p < 0.05$, **$p < 0.01$ *versus* control group by t-test.

C. PTEN-GFP was overexpressed in U87MG PTEN mut cells and G2/M fraction of GFP-positive cells was analysed 72 hours after the treatment. *$p < 0.05$ *versus* control group by t-test.

D. PTEN-GFP was overexpressed in U87MG PTEN mut cells and super G2 fraction, indicating polyploidy, of GFP-positive cells was analysed 24, 48 and 72 hours after the treatment. *$p < 0.05$ *versus* control group by t-test.

Data are represented as mean ± SEM of 3 independent experiments.

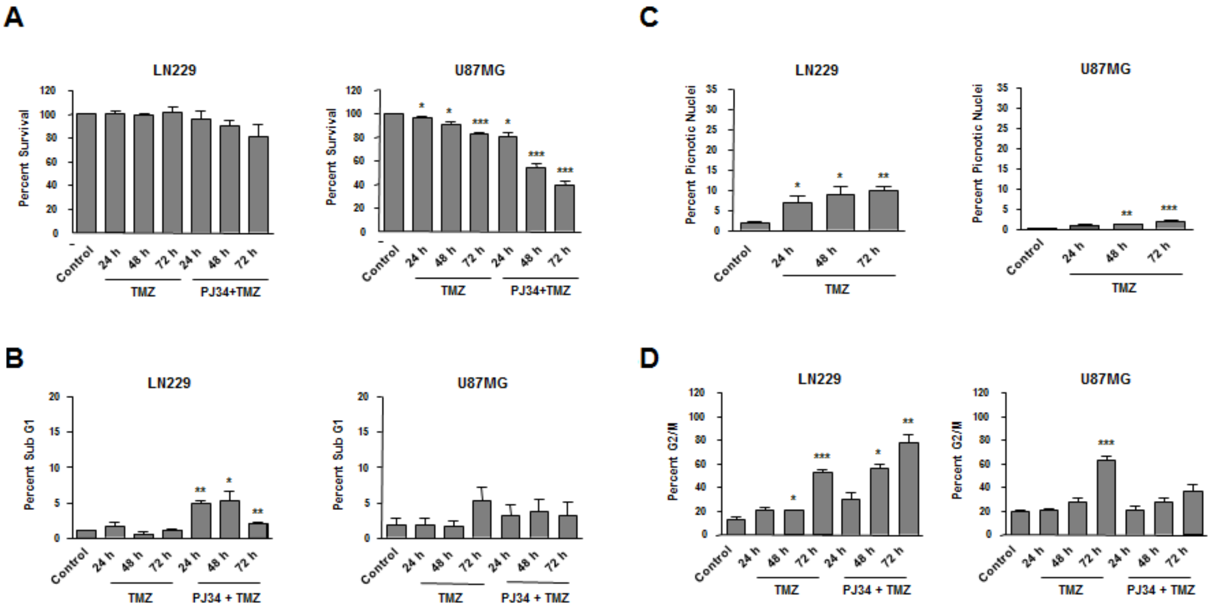

Figure S3: PJ34 do not potentiate Temozolomide (100 µM) effect in LN229 and U87MG glioblastoma.

A. Viability analysis by MTT assay of glioblastoma cells treated for 24, 48 and 72 hours. Data were normalized and expressed as a percentage of the control. *$p < 0.05$, ***$p < 0.001$ by t-test.

B,C Apoptosis activation was determined 24, 48 and 72 hours after the treatment. **(B)** Picnotic nuclei counting (ten fields of view per condition) was performed after the treatments. **(C)** SubG1 fraction was analysed by flow cytometry following staining with PI.*$p < 0.05$, **$p < 0.01$, ***$p < 0.001$ *versus* control group by t-test.

D. Effect of Temozolomide, alone or combined with PJ34, on cell cycle arrest. G2/M fraction was analysed by flow cytometry following staining with PI 24, 48 and 72 hours after the treatment. *p < 0.05, **p < 0.01, ***p < 0.001 versus control group by t-test.

Data are represented as mean ± SEM of 3 independent experiments.

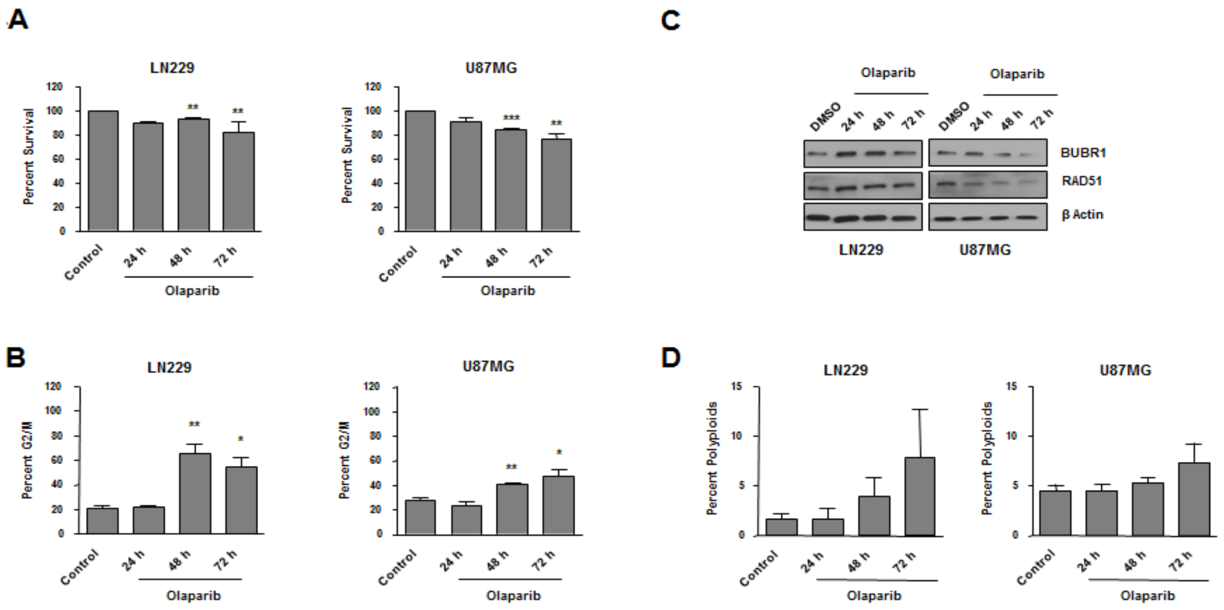

Figure S4: A different PARP inhibitor, olaparib, (10 µM) exerted similar effect to PJ34.

A. Viability analysis by MTT assay of glioblastoma cells treated for 24, 48 and 72 hours. Data were normalized and expressed as a percentage of the control. $**p < 0.01$, $***p < 0.001$ by t-test.

B. Effect of olaparib on cell cycle arrest. G2/M fraction was analysed by flow cytometry following staining with PI 24, 48 and 72 hours after the treatment. $*p < 0.05$, $**p < 0.01$ by t-test.

C. BUBR1 and Rad51 expression was measured by Western Blot 24, 48 and 72 hours after the treatment.

D. Super G2 fraction, indicating polyploid cells, was analysed by flow cytometry after staining with PI.

Data are represented as mean ± SEM of 3 independent experiments.

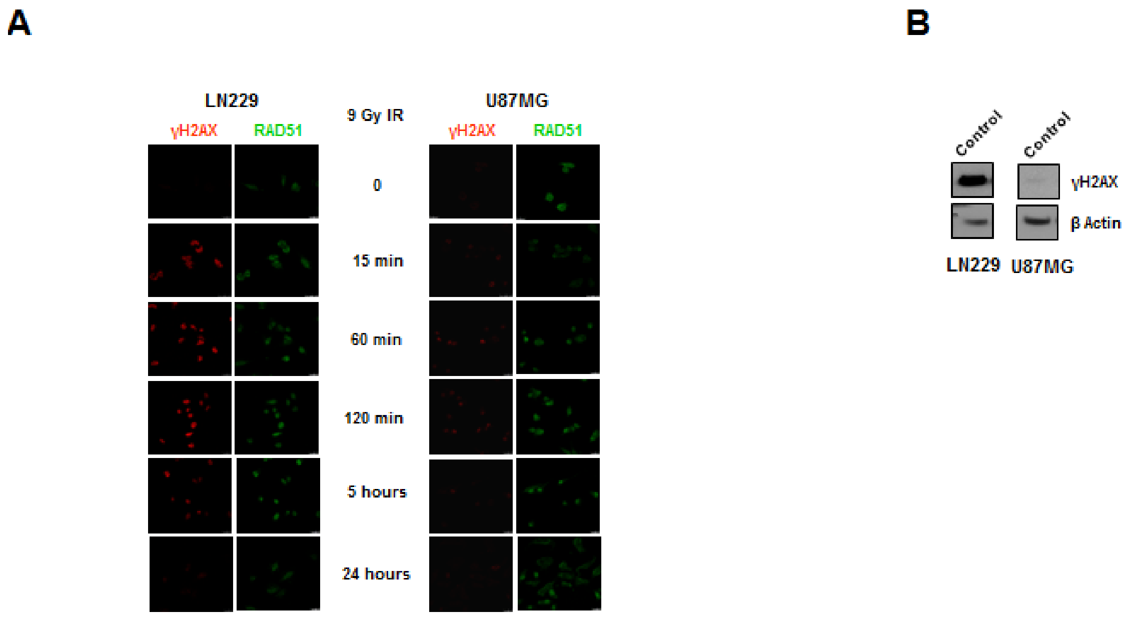

Figure S5: DNA damage signalling is compromised in U87MG cells.

A. Immunofluorescence analysis of γH2AX and RAD51 foci after 9 Gy irradiation. Bar = 25 μM.
B. Western blot analysis of γH2AX basal expression levels.
Data are representative of 3 independent experiments.
